# Service access for youth with neurodevelopmental disabilities transitioning to adulthood: service providers’ and decision-makers’ perspectives on barriers, facilitators and policy recommendations

**DOI:** 10.3389/fpubh.2025.1612509

**Published:** 2025-11-06

**Authors:** Angela M. Senevirathna, Patricia Basualto, Ashish Seth, Gina Dimitropoulos, Jennifer D. Zwicker

**Affiliations:** 1 Faculty of Kinesiology, University of Calgary, Calgary, AB, Canada; 2 School of Public Policy, University of Calgary, Calgary, AB, Canada; 3 Departamento de Kinesiología, Pontificia Universidad Católica de Chile, Santiago, Chile; 4 Faculty of Social Work, University of Calgary, Calgary, AB, Canada; 5 Department of Psychiatry, University of Calgary, Calgary, AB, Canada

**Keywords:** neurodevelopment, transition, access, disability, stakeholders, barriers, facilitators, policy

## Abstract

**Introduction:**

Youth with Neurodevelopmental Disabilities (NDD) who are transitioning to adulthood often struggle with accessing services. This limited access can result in poorer health, reduced ability to perform daily activities and engage in independent living and decreased levels of participation in society. Despite Canada’s commitment to the UN Convention on the Rights of Persons with Disabilities, British Columbia (BC) youth with NDD face additional barriers.

**Methods:**

This study investigated service providers’ and decision-makers’ perspectives on barriers, facilitators and policy recommendations for accessing BC’s health, education and disability services for youth with NDD. We conducted a qualitative descriptive study with 15 semi-structured interviews. We conducted inductive thematic coding to generate themes, which we then organized and interpreted using Bronfenbrenner’s ecological model.

**Results:**

Findings revealed that fragmented organizational structures, eligibility criteria, limited and unstable funding and enduring stigma impede service access, while coordinated inter-agency collaboration, clear transition planning and early, family-centered interventions may improve outcomes.

**Discussion:**

Targeted policy reforms across multiple ecological levels are essential to reduce inequities in service access and strengthen the continuum of support for youth with NDD as they transition to adulthood.

## 1 Introduction

Youth with neurodevelopmental disability (NDD) are a heterogeneous group with chronic conditions that originate during the developmental period affecting the central nervous system, resulting in impact on daily functioning (1). In Canada, an estimated 75% of youth with a disability have a NDD, with prevalence ranging from 5 to 9% and up to 15% in developed countries (2–4). Youth with NDD often encompass conditions affecting their cognition, communication, motor skills, social interaction and behavior which leads to barriers in full participation in society (5). Moreover, youth with NDD often require extensive healthcare and support services and require continuous support as they transition into adulthood (6, 7).

The transition to adulthood is a crucial developmental phase focused on emotional, psychosocial growth and independent living (8), requiring youth to establish new relationships with service providers and navigate new systems of care (9). In Canada, it involves the transition from pediatric to adult healthcare, special education to adult life and a shift from pediatric to adult disability supports, as well as caregiver to self-income supports (10, 11). This critical phase is often characterized as navigating a “support cliff” (12), leading to a considerable decrease in support service utilization after reaching adulthood (13).

There are considerable gaps in the availability and quality of transition service systems for youth with NDD. These challenges include poor communication between pediatric and adult services, limited resources, a lack of understanding about transition practices among adult care providers, insufficient planning for transitions and the anxiety young adults feel when facing new healthcare systems (9, 14).

While challenges in the transition from pediatric to adult services have been widely discussed (9–11), little is known about how these factors operate across systems simultaneously. Youth with NDD often encounter poorly coordinated care and service gaps at the intersections of health, education and social services (15). Much of the existing literature remains siloed, addressing barriers within a single domain. For example, inadequate preparation for transfer in healthcare or transition tools developed for one sector (16). A smaller body of work demonstrates the value of cross-system perspectives. Hoffman et al. applied Bronfenbrenner’s ecological model to parents’ accounts of transition experiences for autistic youth, showing influences at multiple levels (17). Similarly, Mirzaian et al. mapped the “bouncing” of young people with intellectual and developmental disabilities between disability and mental health systems, illustrating how multi-level barriers accumulate (18). Despite these advances, cross-system analyses remain rare, and most studies continue to conceptualize barriers as isolated rather than interdependent processes. This gap also extends to understanding the unique needs of youth with NDD, strategies for supporting their transition and the systemic and organizational barriers that hinder successful outcomes (18). The consequences of these service gaps are severe: youth with NDD face higher risks of homelessness, greater involvement in the criminal justice system, lower graduation rates, reduced participation in post-secondary education, limited workforce engagement and higher rates of poverty than peers without disabilities (19–21). These findings highlight the urgent need for coordinated, multi-system approaches to ensure appropriate support for youth with NDD transitioning to adulthood.

To address this gap, we draw on Bronfenbrenner’s ecological systems theory as a framework (22). Transitions across service systems are not shaped by isolated factors but by the interaction of influences spanning multiple levels. At the microsystem level, youth and families navigate daily encounters with clinicians, educators and case managers; at the mesosystem level, interactions between schools, health providers and community agencies determine whether supports are aligned or fragmented; at the exosystem level, organizational rules and service delivery structures often create barriers; and at the macrosystem level, provincial funding schemes and eligibility criteria establish the policy barriers for access (22). Unlike narrower health transition frameworks, the ecological model explicitly foregrounds how these nested layers interact over time, a perspective particularly suited to the complex, cross-sectoral nature of transitions for youth with NDD (17, 18). This framing allows us to situate qualitative accounts of transition within a multi-level policy context, ensuring that findings not only capture lived experience but also illuminate the structural dynamics that either constrain or enable access to adult disability supports.

Service providers and decision-makers are important key informants regarding barriers and facilitators to accessing services when transitioning from pediatric to adult supports. Through daily engagement with policy and program implementation, service providers and decision-makers come to know what works and under what conditions (23). By centering service providers and decision makers, we illuminate the implementation layer of transition policy: how eligibility rules are operationalized, how organizational constraints drive triage and wait listing, and how inter agency relationships either enable or obstruct continuity.

Therefore, this paper focuses on describing decision makers’ and service providers’ perspectives in British Columbia, Canada to better understand barriers and facilitators in accessing services for youth with NDD based on two core research questions:

RQ1: How do barriers and facilitators operate across Bronfenbrenner’s ecological levels (micro, meso, exo, macro, chrono) to shape service access during transitions from pediatric to adult services for youth with NDD in British Columbia?

RQ2: Which multi-level policy levers can reduce inequities and improve service continuity during the transition to adulthood?

The aim is to describe the barriers and facilitators within systems and how they influence one another, so that interventions and support services can be tailored to facilitate a smooth transition for youth with NDD, promoting their successful integration into adult life. We apply Bronfenbrenner’s ecological systems theory as an organizing lens to map inductively derived themes across micro-, meso-, exo-, macro- and chrono-levels and to surface cross-level interactions. Findings from one provincial jurisdiction in Canada provide important context for other systems, including policy implications, resource allocation and the coordination of services.

## 2 Materials and methods

### 2.1 Study design

This study utilized a qualitative research design to gain a deeper understanding of the perspectives service providers and decision-makers have on barriers and facilitators that youth with NDD encounter in the transition from pediatric to adult services. Semi-structured interviews were employed to capture participants’ perspectives, grounded in their firsthand experiences working within systems serving youth with NDD and their families. This design allowed for an exploration of nuanced insights from those directly involved in the delivery of services and system design.

We adopted a qualitative descriptive approach within a pragmatic paradigm. Inductive coding generated themes that were subsequently organized using Bronfenbrenner’s ecological model (24, 25). This approach was well-suited to capture the range of experiences, challenges and facilitators perceived by service providers and decision-makers from various fields, including health, special education and disability supports (25). The research design was part of a broader, multi-method project focused on exploring service access for children and youth with NDD. Ethical approval for the study was obtained from The University of Calgary’s Research Ethics Board (REB20-1872).

### 2.2 Participant recruitment

Participant recruitment took place from January 2023 to November 2023. A combination of purposeful and snowball sampling methods was used to identify and recruit participants (26). We used purposeful sampling to recruit service providers and decision-makers with direct experience in supporting youth with NDD during the transition from pediatric to adult services. This approach ensured participants had situated knowledge of the systems and policies under study (26). Snowball sampling was also employed, as participants were well-positioned to identify additional colleagues across health, education and social service sectors whose perspectives were essential for capturing the cross-system nature of transitions (26). To be eligible, participants had to meet the following inclusion criteria: they were either actively working in a decision-making or service provider role in health, special education or disability services at the time of data collection, or they had previous experience with organizations providing services and supports to youth with NDD transitioning to adulthood in British Columbia. Eligible participants who expressed interest were invited by the researchers via email to participate in interviews. The email included a link to the informed consent form and a brief demographics survey to gather participant characteristics. Once the completed consent form was received, participants were provided with a link to join the interviews via Zoom Video Conferencing Software.

### 2.3 Data collection

Semi-structured interviews was the primary method of data collection for this study. The interview guide was developed based on previous research (27), which explored the experiences of children with NDD and their families in accessing services across Canada. The interview guide included questions regarding eligibility criteria, barriers and facilitators to accessing services and supports, changing needs and waitlists (Supplementary material A). The guide was pilot tested with a service provider and subsequent modifications were made to enhance its quality and clarity.

Feedback on the interview questions was obtained from the multidisciplinary research team and the advisory council, which included individuals with lived experience, knowledge users and community partners, to ensure the questions were appropriate and comprehensive. Interviews were conducted virtually using Zoom Video Conferencing, were audio-recorded and transcribed verbatim using ReV software. Each interview lasted up to 90 min and involved one interviewer and one note-taker. The audio recordings were stored in a secure encrypted drive provided by the University of Calgary. A total of 15 interviews were conducted (nine service providers and six decision-makers), representing both urban and rural communities across the province of British Columbia. Participant characteristics are outlined in Table 1.

**Table 1:** Participant characteristics.

Participants	Program/Service	Gender Man (Woman)	Age ranges	Communities served
Service providers	Health	2 (0)	(55–64): 2	Rural: 1 Urban & Rural: 1
	Education	0 (1)	(45–54): 1	Urban:1
	Social service	3 (3)	(35–44): 1 (55–64): 4 (65–74): 1	Rural: 2 Urban & Rural: 4
Decision Makers	Health	1 (0)	(55–64): 1	Urban & Rural: 1
	Education	1 (0)	(55–64): 1	Urban: 1
	Social service	2 (2)	(45–54): 2 (55–64): 4	Urban: 1 Urban & Rural: 3

### 2.4 Data analysis

The qualitative data analysis followed a six-stage thematic analysis process; familiarization with the data, generating initial codes, searching for themes, reviewing themes, defining and naming themes and writing the final report (28). Ten transcripts were read multiple times to gain familiarity with the data. We then conducted initial line-by-line inductive coding, staying close to participants’ words to capture barriers, facilitators and transition experiences as they were described. The iterative codebook approach was used to ensure consistency in coding across interviews (Supplementary material B). Then the codebook was applied to all transcripts, with ongoing comparison and refinement. New inductive codes were added when data did not align with existing categories. Codes were clustered into broader sub-themes that described patterns across participants’ accounts. These inductively derived sub-themes were then mapped to ecological levels to locate each theme within the model (organization/interpretation), not to generate themes. The deductive analysis offered a structured way to situate participants’ accounts within a rigorously theorized framework, ensuring system-level mechanisms were captured alongside lived experiences. Post-coding ecological mapping provided a structured way to organize and interpret participants’ accounts. This hybrid approach of inductive/deductive thematic analysis ensured that lived experiences remain central while also informing policy and organizational responses (29). This approach enabled the researchers to capture emergent insights while structuring interpretation through the ecological framework (29, 30).

**Figure 1:**
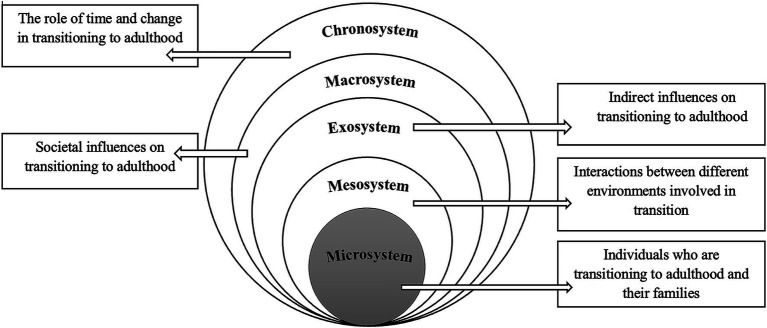
Interactions between systems using Bronfenbrenner’s ecological model to organize and interpret inductively derived themes.

The deductive analysis of the data was situated in Bronfenbrenner’s ecological systems theory (22), which provided a conceptual framework for understanding the transition of youth with NDD into adulthood. This model emphasizes the complex interactions between individual and social and systemic factors, including the microsystem, mesosystem, exosystem, macrosystem and chronosystem. Youth and families navigate not only immediate clinical or educational encounters but also organizational policies, professional cultures and interagency relationships, as well as broader provincial funding structures and eligibility criteria. By applying this model, we critically examine how multi-layered environments interact to produce both barriers and facilitators for service access. Unlike alternative frameworks that focus narrowly on health care delivery or individual behavior, the ecological model foregrounds the cross-system interactions that are central to policy design in disability services (Figure 1). However, the analysis was conducted within a qualitative descriptive design; while inductively generated themes were mapped to ecological systems theory, the study was not theory-driven.

Initial coding was led by the doctoral researcher, with supervisory team members independently reviewing a subset of transcripts to refine the coding framework. Differences in interpretation were discussed in team meetings until consensus was reached.

As qualitative research is shaped by researchers’ social positions, disciplinary training and prior experiences, reflexivity was practiced as an ongoing team process. The team brought complementary perspectives of health policy and health-economics expertise (supervisory role), clinical and social work practice expertise in child and youth navigation (supervisory role) and a doctoral researcher in health policy with ongoing training in disability policy and service access. These backgrounds, including prior collaborations with disability service systems, oriented us toward system and policy-level interpretations and an emphasis on equity. To mitigate bias, two researchers independently reviewed the transcripts to become familiar with the data and generate codes, with oversight and support from the qualitative lead. Initial findings were presented to senior researchers for peer debriefing to enhance the rigor and credibility of the analysis (31, 32). Following the coding process, the researchers exchanged transcripts and collaboratively resolved any discrepancies, ensuring validity through consensus (33). Themes were then developed from the codes and group discussions were held to refine these themes (34). Insights from these discussions were documented to maintain a transparent and rigorous process.

The final sample of 15 interviews was considered adequate to answer the aim and research questions based on the concept of “information power” (35), which suggests that the data provided sufficient depth and breadth for meaningful conclusions. While the final sample of 15 interviews may appear modest, sufficiency was demonstrated by the recurrence of central themes across participants and the redundancy of perspectives after approximately 12 interviews. Importantly, the sample included a range of roles and organizations, which provided variation in context while still allowing thematic convergence. This aligns with qualitative guidance that emphasizes adequacy of information richness and diversity of perspectives over numerical thresholds (35, 36).

To further ensure the validity and relevance of the findings, preliminary results were shared with the advisory council for feedback, ensuring alignment with the perspectives of those familiar with the topic (31–33).

## 3 Results

### 3.1 Demographics

A total of nine service providers and six decision-makers were selected and interviewed for the study. Participant characteristics reflected a diverse range of perspectives, including variation across provider type, ministry type, age and service area (Table 1).

### 3.2 Findings

**Figure 2:**
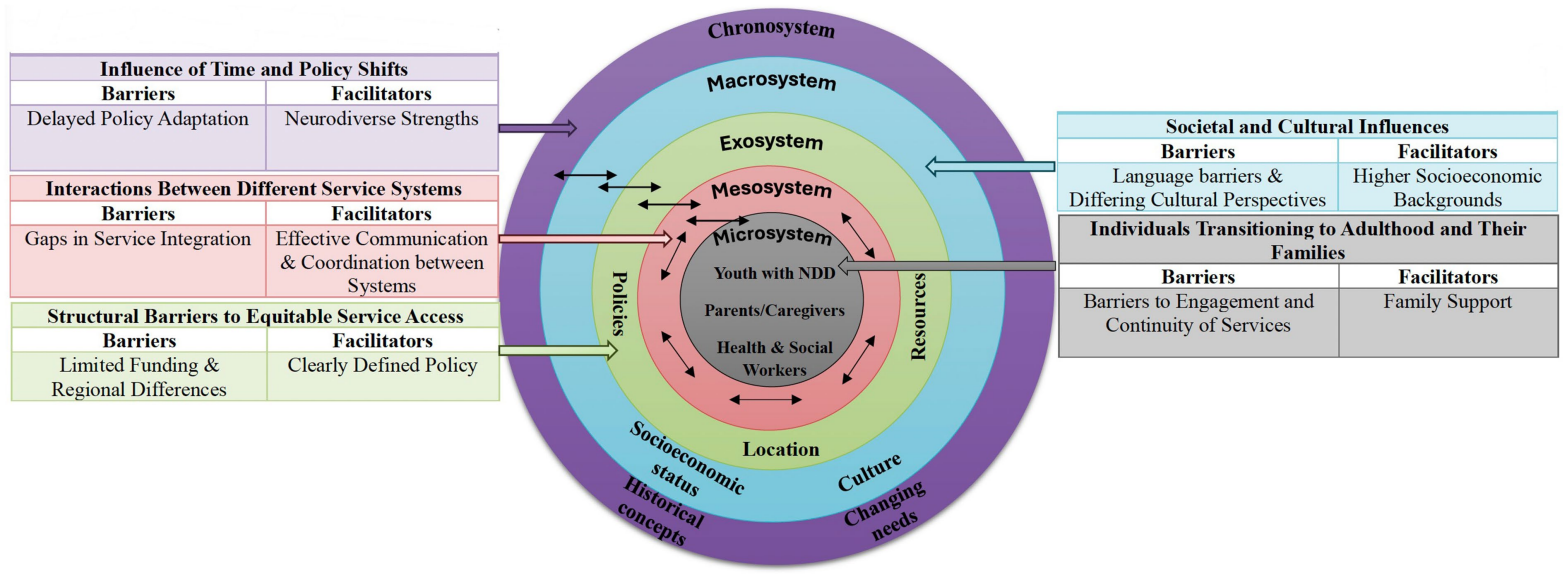
Themes and subthemes.

Inductively derived themes were organized and interpreted using Bronfenbrenner’s ecological model to examine how barriers and facilitators interact across systems. We identified key influences at multiple ecological levels, highlighting the complex interplay between individual, family, service and policy factors that affect service access and continuity. The findings included a series of policy recommendations aimed at improving service delivery, increasing equity in access and enhancing coordination between systems. Figure 2 provides an overview of the findings. Quotes are presented as illustrative evidence embedded within analytic text, consistent with qualitative description. Headings by ecological level reflect organizational choices; themes were derived inductively from the data.

#### 3.2.1 Microsystem: individuals transitioning to adulthood and their families

At the microsystem level, youth with NDD and their immediate support networks, including family members, caregivers and healthcare providers, play a pivotal role in shaping access to services. Family support emerged as an existing critical facilitator in navigating service systems for some youth. Parents and caregivers often acted as advocates, securing necessary services and ensuring continuity of care. However, barriers to engagement and continuity were prominent. Youth frequently disengaged from support services due to stigma and a lack of understanding of their future needs:

“The older the youth with support needs, they do not feel they need the support [.] they do not want to be labeled disabled” (SP10).

Families who were unfamiliar with transition planning faced challenges in identifying and accessing adult supports. For instance, in British Columbia, without proper guidance, the adult support system can be complex and daunting for families and youth seeking support and the intricacies of application processes and available services can be difficult to understand and navigate:

“One of the biggest and scariest things for (our) families is the persons with disabilities application” (SP06).

#### 3.2.2 Mesosystem: interactions between different service systems

The mesosystem reflects the interactions between various support systems, including pediatric and adult service providers. Effective communication and coordination between these systems were key facilitators for smooth transitions. However, participants identified significant gaps in service integration and coordination, resulting in fragmented care and service discontinuities. For example, the shift from Children and Youth with Special Needs (CYSN) services to adult services like Community Living British Columbia (CLBC) was not always seamless:

“CLBC relies on other systems to create access pathways to ours, but those systems do not know sometimes that we are relying on them” (DM04).

According to participants, the current attempts to improve collaboration between systems include the creation of internal resources to enhance service literacy and promote coordinated care. One participant described that they developed an internal webpage with information on CLBC to help staff better understand its scope and limitations, noting that:

“it’s our effort to try and increase literacy on these services within our organization” (SP02).

Despite these efforts, siloed service structures with rigid eligibility criteria continued to pose barriers for families:

“It’s a very rigid criteria. You either are or are not eligible” (DM04).

#### 3.2.3 Exosystem: structural barriers to equitable service access

At the exosystem level, decisions made by government agencies and policymakers indirectly influenced service access. The policy for CLBC’s eligibility is clearly defined, an individual qualifies if a psychologist determines they meet the requirements:

“You (either) meet the threshold requirements and have a developmental disability, autism or FASD you are then eligible to receive services from CLBC” (DM08).

Structural barriers, including limited funding and regional differences in service availability, were key challenges. Participants described financial constraints that limited-service provision and created lengthy waitlists:

“CLBC has a finite budget [.]. So we try to manage within the parameters and then prioritize individuals who have the greatest needs” (DM02).

Geographic location also created disparities in access, with youth in rural and remote communities facing additional barriers. Participants highlighted that even when individuals were deemed eligible for CLBC, services were often unavailable in isolated regions. In some cases, communities could only be reached by air for part of the year, leaving families without consistent access to supports.

“Not just rural that you can drive to, but remote where it’s fly in, fly out for part of the year and there may or may not be any services in the community at all” (DM04).

#### 3.2.4 Macrosystem: societal and cultural influences

The macrosystem encompasses broader societal values and socioeconomic factors that shape service access. Families from higher socioeconomic backgrounds were better able to navigate service systems and secure necessary supports:

“People with intellectual disabilities who are born into affluent privileged families have by far a better situation, receive better services, have better housing” (SP04).

Cultural beliefs and practices also influenced service utilization, with immigrant families encountering language barriers and differing cultural perspectives on disability. One participant explained that service use often depended on how disability was understood within a family’s cultural background and in some cases, those perspectives could discourage engagement:

“it depends on your cultural background and maybe those perspectives on disability and sometimes that might prevent you from accessing” (SP08).

#### 3.2.5 Chronosystem: influence of time and policy shifts

The chronosystem reflects how transition experiences evolve over time in response to policy changes and shifting societal norms. There was increased recognition of the strengths and contributions of neurodiverse individuals to society, leading to a shift toward more inclusive models of support:

“Kids that are transitioning into adulthood, we are seeing changing needs as we become more aware of the strengths and contributions that neurodiverse people can make to society” (SP08).

Historical comparisons further underscored the scale of change. Whereas past systems often segregated individuals with NDD from community life, participants noted a shift toward inclusive approaches:

“Young people are included in schools in a way that they were not 30 years ago, so a much greater need to just be part of the community, to get away from the more segregated models of support to much more inclusive models” (SP04).

Consistent with our design, these observations reflect participants’ perceptions rather than longitudinally demonstrated trends.

#### 3.2.6 Policy and system level implications

Participants emphasized several policy priorities to strengthen transitions for youth with NDD. A consistent theme was the importance of case management to ensure continuity, with one noting that:

“Case management would be a helpful thing [.] you need that one person to help hold the story as they go from child and youth to adults” (SP02).

In addition, stronger collaboration between systems was viewed as essential. It was suggested that CLBC could improve:

“How, when and who we engage with [and] pursue it more actively than historically” (DM04).

Gaps in regional coordination were also raised, with participants pointing out that:

“CLBC has missed that middle gap[.] we need to be in the local networks, but also in the regional network” (DM04).

Resourcing was another prominent theme, with participants stressing the need for expanded capacity to support adults:

“It would be great if we could increase the ability to support adults” (SP08).

Equity concerns further underscored the importance of targeted outreach, as participants described the need for:

“Making sure organizations are aware of our services” (DM01).

Finally, participants identified emerging needs such as co-occurring substance use, describing this as:

“An emerging area” (DM02).

Requiring greater attention.

## 4 Discussion

### 4.1 Summary of findings

The transition from youth to adulthood is a formative time for everyone, but for individuals with NDD, it presents unique challenges. This study highlights how service providers’ and decision-makers’ perspectives reveal individual and systemic barriers and facilitators in the transition from pediatric to adult services for youth with NDD. Additionally, the findings offered policy recommendations aimed at improving transition services and addressing these challenges. In our analysis, the perspectives of service providers and decision-makers were largely convergent, emphasizing common priorities such as the need for case management, stronger interagency collaboration and increased resourcing to support transitions. Service providers more frequently highlighted the day-to-day challenges of navigating fragmented systems and supporting families with limited resources, while decision-makers focused on structural issues such as policy gaps, regional coordination and strategic planning. Despite these differences in emphasis, divergences were minimal.

Previous research has consistently documented the barriers that youth with NDD encounter when transitioning to adulthood. Studies highlight how stigma (37) and lack of support networks (37, 38) discourage service engagement, leading many young people to disengage from needed supports and experience social isolation. The transition is further complicated by simultaneous changes in healthcare, education and social networks, alongside heightened expectations for independence (9, 38). Socioeconomic resources play a crucial role in shaping outcomes. Families with financial means are better able to secure assessments, consultants and timely services, while lower-income families face systemic disadvantages (37, 38). Rigid diagnostic criteria exacerbate these inequities, excluding youth whose needs do not align neatly with established categories, especially those with overlapping or co-occurring conditions (39, 40). Fragmentation between pediatric and adult systems, often operating in silos, compounds these challenges and undermines continuity of care (23, 41, 42). Structural barriers, such as limited funding, is found to be another barrier for accessing services (43). Geographic disparities further limit access, as youth in rural and remote communities have fewer specialized services compared to their urban peers (13, 44). Finally, cultural and linguistic barriers disproportionately affect immigrant families, who may face stigma within their communities or lack familiarity with service systems, leaving them less likely to seek formal support (45, 46). While prior research has underscored some barriers, our findings extend this work by showing how these dynamics emerge not in isolation but through interactions across ecological levels.

Bronfenbrenner’s ecological model was a useful way to organize our findings. The model treats systems as layers that surround the individual, yet our results show these systems are not neutral. They actively shape who gets access to services and who is left out. Rules about eligibility, funding decisions and how services are organized across regions act as gatekeeping tools that can exclude people (47). As Prince (2012) argues, disability policies often follow neoliberal logics, approaches that prioritize efficiency, cost-cutting and individual responsibility over equity and inclusion, which deepens marginalization (48). Participants’ accounts suggest processes through which exclusion may be produced through how systems interact.

First, the participants described how youth disengagement at the microsystem level, often shaped by stigma and lack of family support, and intersects with rigid eligibility rules at the exosystem level and broader societal inequities at the macrosystem level, producing compounded exclusion. Second, it showed that failures in interagency collaboration at the mesosystem level cannot be separated from resource scarcity and political priorities at the exosystem level. Fragmentation reflects not only weak communication but structural underfunding. Third, it applied the ecological framework to situate participants’ accounts, highlighting that systems are not neutral contexts but sites of governance and power, where eligibility rules, budgetary decisions and geographic structures function as gatekeeping mechanisms (46–48).

### 4.2 Inter-level interactions

Participants described disengagement, which they linked to older teens resisting being “labeled disabled” (49). This disengagement intersected with rigid exosystem eligibility rules that required categorical diagnoses. Youth who resisted services thus faced double exclusion, first by stepping away and second by systems with rigid eligibility pathways. Previous research has shown similar dynamics where stigma drives youth to avoid disability labels, while rigid eligibility criteria act as structural gatekeepers (50). Cheak-Zamora et al. describe this as the “service cliff,” where young people simultaneously disengage and age out (51). Our findings reinforce that stigma is not only interpersonal but institutionally reproduced, as systems fail to provide flexible, youth-centered re-engagement options.

The interviews revealed the overwhelming complexity of disability benefit applications for youth and families. These microsystem-level burdens were intensified by the absence of case management or consistent supports across the systems. Without a single point of contact, families carried responsibility for coordination, increasing stress and risking youth disengagement. Prior studies show that the absence of navigation supports disproportionately disadvantages families with fewer resources (52). Evidence from Canada and internationally indicates that case management improves continuity and outcomes by ensuring that one actor “holds the story” during transitions (53–55). Our findings align with this literature, underscoring that the absence of mesosystem supports transforms administrative complexity into structural exclusion.

The lack of interagency collaboration (mesosystem) was linked to broader exosystem constraints. Participants described finite budgets that forced prioritization of “greatest needs,” limiting capacity for proactive collaboration. Thus, fragmentation was not only a product of communication failure but also the political economy of scarcity. Previous studies confirm this link. Beresford et al. observed that service fragmentation reflected resource shortages, while US studies highlight how underfunded systems foster agency competition rather than collaboration (56, 57). Canadian evidence similarly shows that collaboration frameworks require not only will but also resources and accountability (16). Our findings suggest that mesosystem collaboration cannot succeed without exosystem investment.

Rigid eligibility criteria and funding limits (exosystem) interacted with macrosystem inequities of socioeconomic and cultural factors (58). Affluent families secured private assessments and leveraged social networks, while immigrant families faced linguistic barriers and cultural stigma (59). Research across international contexts shows that socioeconomic privilege translates into smoother navigation (60, 61), while immigrant families encounter systemic barriers tied to cultural framings of disability (58). Canadian research argues that disability systems reproduce broader inequities by rewarding those with financial and cultural capital (62, 63). Our findings reinforce that eligibility and funding rules are not neutral and they interact with macrosystem inequities to stratify outcomes.

Geographic disparities (exosystem) further illustrated inter-level entanglements. Families in remote communities were eligible but had no services available locally (64). Here, exosystem structures (lack of regional delivery models) intersected with macrosystem inequities (urban-centric policy design) and translated into microsystem struggles for families left unsupported. Australian research on the NDIS documents similar rural service gaps (65) and Nordic countries face professional shortages in rural areas despite universalist policies (66). These findings highlight that geography is not only logistical but also reflects structural decisions about resource distribution.

Furthermore, emerging challenges such as co-occurring substance use revealed how chronosystem shifts in population needs collide with static exosystem rules. Eligibility frameworks designed in the early 2000s had not adapted, leaving families navigating disconnected disability and mental health systems. Participants also noted evolving societal understandings of neurodiversity, showing how macrosystem values change more slowly than youth realities. Studies of policy inertia confirm that adolescent mental health and co-occurring conditions are poorly accommodated in static service frameworks and transitions fail when systems cannot adapt to shifting needs (51, 53, 62, 67). Our findings add that time itself becomes a site of exclusion. Participants perceived that policy lag can leave youth unsupported in evolving contexts.

However, some considerations need to be undertaken. Although Bronfenbrenner’s ecological systems theory offers a valuable lens for structuring multi-level influences on transition, it does not fully capture structural exclusion or the ways inequities are reproduced through policy design, eligibility criteria and socio-political ideologies. Other frameworks, such as Andersen’s behavioral model of health services use (68) and the Life course health development (LCHD) model (69), emphasize the dynamic interactions between individual characteristics, service systems and broader social determinants of health. These perspectives foreground how system level characteristics such as policies, healthcare facilities and personal characteristics such as education, social background, shape service access, particularly for marginalized youth.

The interactions identified in this study such as how resource shortages intersect with cultural barriers and restrictive eligibility policies, illustrate that barriers cannot be understood in isolation. Instead, they accumulate and interact to create compounded exclusion over time, reinforcing the importance of examining transitions as embedded in intersecting systems rather than discrete challenges. By situating our findings in relation to these broader frameworks, we underscore the unique insight of this study. That transition barriers for youth with NDD emerge not only from isolated characteristics but also from the interaction of ecological levels with structural inequities, highlighting the need for integrated, equity-oriented policy reform.

### 4.3 Study limitations

This study’s strength lies in capturing perspectives across multiple systems, offering insight into how policy is enacted in practice. The ecological framework facilitated analysis of inter-level interactions, though it required adaptation. Limitations include the BC-specific sample, which may limit transferability and the absence of youth and family voices, which are critical for future research. Although purposive and snowball sampling enabled access to a diverse group of providers and decision-makers, we acknowledge that these approaches may have over-represented individuals who are well connected within service networks and under-represented dissenting or marginalized voices (70). We sought to mitigate these risks by recruiting through multiple sectors and organizational levels, but we recognize that the absence of certain perspectives remains a limitation that should temper the transferability of our findings. At the same time, the recurrence of key themes across participants and the breadth of organizational vantage points represented provide confidence that the study identifies system-level patterns and policy-relevant mechanisms that are unlikely to hinge on a single perspective. Because data were cross-sectional, chronosystem inferences reflect participants’ retrospective accounts (e.g., policy shifts, evolving norms) rather than longitudinal observation.

### 4.4 Practice recommendations

Policy recommendation 1: implement person-centered transition planning with case management supports.

Transition planning for youth with NDD should move beyond diagnostic and IQ thresholds to emphasize functional abilities, strengths and personal goals. In BC, this could be advanced by strengthening CLBC’s cross-ministerial protocol for transition planning for youth with special needs (71). Embedding case managers within CLBC regional offices would provide families with a single point of contact and improve continuity across education, health and social care as evidence from Alberta’s transition planning initiatives and international studies supports case management as a way to reduce fragmentation and promote smoother transitions (72).

Policy recommendation 2: establish proactive interagency collaboration frameworks.

Systemic barriers in the transition process are often reinforced by fragmented communication and unclear responsibilities between pediatric and adult service systems (73). A proactive collaboration framework should outline when, how and by whom engagement occurs across agencies, ensuring that responsibility for information sharing does not fall passively on individual families or disconnected systems (74). By embedding active mechanisms for interagency coordination, rather than relying on *ad hoc* efforts, such a framework would reduce service gaps, promote consistency in communication and strengthen continuity of care for youth with NDD.

Policy recommendation 3: develop regional support networks to strengthen rural service delivery.

Geographic disparities remain a significant barrier for families in rural and remote parts of BC, where services are often sparse or unavailable. Establishing regional support networks would bridge the “middle gap” between provincial structures and local providers, embedding CLBC fully into both local and regional systems (75). Frameworks for rural health care planning emphasize community-specific approaches, multidisciplinary team-based care, expanded use of telehealth, systematic evaluation and strategies to recruit and retain providers (76). Incorporating these principles into disability service planning would improve outreach, expand availability and ensure families in remote communities have more consistent access to supports.

Policy recommendation 4: increase funding for transition services and housing supports.

Resource constraints limit service availability, generate long waitlists and deepen inequities for youth and families. In BC, expanding funding through targeted streams such as CLBC’s personalized supports initiative and partnerships with BC housing’s supported independent living programs would help ensure that transition planning and housing supports are not delayed or inaccessible (77). Dedicated allocations would allow agencies to expand service options and reduce inequities in access, particularly for low-income and underserved populations (47, 71).

Policy Recommendation 5: Strengthen outreach and culturally responsive supports for marginalized communities.

Immigrant families and other marginalized groups often face language barriers, stigma and cultural perspectives on disability that limit service uptake (78). In BC, stronger partnerships between CLBC and community-based organizations, including settlement and integration services, could expand outreach and ensure families are informed about available supports. Embedding cultural competence and linguistic accessibility into provider training would reduce barriers, build trust and promote more equitable access.

Policy recommendation 6: implement a complex care model to address co-occurring needs.

Youth with NDD are increasingly presenting with emerging challenges such as substance use and mental health concerns, yet current transition structures remain siloed and ill-equipped to respond (79). Developing a complex care model that integrates disability, mental health and addictions services would ensure these needs are addressed within the transition process (80). In BC, this could build on initiatives such as the Integrated Child and Youth Teams (77) being rolled out across the province, expanding their scope to include developmental disability supports. Embedding multidisciplinary expertise and coordinated pathways into such models would reduce fragmentation and provide more responsive, holistic care.

### 4.5 Conclusion

This study shows that transition barriers for youth with NDD are not isolated but produced through interlocking dynamics such as stigma, eligibility rigidity, resource scarcity, inequities of privilege and culture, geographic divides and policy. We use the ecological model to synthesize participant perspectives on inter-level interactions and we situate BC’s findings within international debates on disability policy. We intentionally kept the practice recommendations section distinct, to ensure that these recommendations were presented as a synthesis of participant perspectives.

Our findings underscore that reforms targeting single levels are insufficient. Anti-stigma programs cannot succeed if eligibility remains rigid. Additional funding will not resolve inequities if services remain siloed. Outreach to immigrant communities will fail if eligibility and geographic barriers remain unchanged. And static frameworks cannot meet the needs emerging over time. Addressing these barriers requires integrated, cross-level reforms that are person-centered, equity-focused and adaptive to emerging needs. In doing so, disability transition policies can move beyond fragmented, exclusionary models toward inclusive pathways to adulthood.

## Data Availability

The datasets generated or analyzed during the current study are not publicly available due to the confidential nature of qualitative interview data and the ethical obligations to protect participant privacy. Participants did not consent to the public sharing of their transcripts. Requests for de-identified excerpts may be considered on a case-by-case basis and subject to approval by the appropriate research ethics board. Requests to access the datasets should be directed to angela.senevirathna@ucalgary.ca.

## Supplementary material

SUPPLEMENTARY TABLE 1 Summary of themes, subthemes, illustrative codes and example participant quotations identified through qualitative analysis of service provider and decision-maker interviews. This table outlines the analytical structure supporting the key findings.

SUPPLEMENTARY DATA SHEET 1 Interview guide.
