# Supplementary material for: Service access for youth with neurodevelopmental disabilities transitioning to adulthood: service providers’ and decision-makers’ perspectives on barriers, facilitators and policy recommendations
Source: Front Public Health. 2025 Nov 6;13:1612509. doi: 10.3389/fpubh.2025.1612509 (PMC12631288; doi:10.3389/fpubh.2025.1612509)
Supplement: SUPPLEMENTARY TABLE 1 — Summary of themes, subthemes, illustrative codes and example participant quotations identified through qualitative analysis of service provider and decision-maker interviews. This table outlines the analytical structure supporting the key findings. [file Table_1.docx]

| **Theme** | **Sub-theme** | **Code** | **Code description** | **Example quote** |
| --- | --- | --- | --- | --- |
| Microsystem: Individuals Transitioning to Adulthood and Their Families | Family specific challenges | Challenges for families | Barriers that families face before accessing services | Without support to connect individuals to services, they risk falling through the cracks and not receiving needed care (DM2). |
|  |  | Eligibility barriers for families | Barriers families face when considering eligibility for programs | One individual’s cognitive impairment worsened with age, but because he didn’t meet the threshold before 19, he’s ineligible for services after 19 (DM4). |
|  |  | Accessibility barriers for families | Barriers families face when accessing the eligible programs | Support for transitioning to adult services, programs, and independent living is very minimal (SP15). |
|  | Challenges in transition | Individual level challenges | Barriers that families face in transition at individual level | The older the youth with support needs, they don't feel they need the support [..] they don't want to be labelled disabled (SP10). |
|  | System navigation and  Understanding | Awareness | Awareness of families about the support systems | I am seeing challenges, even in family's ability to understand how to negotiate services with them [SP2]. |
|  |  | Navigating the system | How do families go through the process of looking for available services | You have to have a social worker, a doctor, and support network, fill out this copious amount of paperwork to qualify for persons with disability benefit [SP6]. |
|  |  | Understanding the system | General understanding of families about the systems in place | Those are the people who have the easiest access, [..], these are parents who understand and have access to the right assessments (DM4). |
| Mesosystem: Interactions Between Different Service Systems | Organizational challenges | Challenges for organizations | Barriers the organizations face when they provide services to the families | There are historical challenges between health authorities and CLBC, though guidelines for collaborative service delivery exist (SP02). |
|  |  | Eligibility criteria barriers | Barriers that organizations face when considering eligibility for programs | It’s a very rigid criteria. You either are or are not eligible (DM04). |
|  |  | Barriers in assessments | Barriers organizations face when assessing the conditions of the families | We have little access to health data, so we don’t know clients’ hospital use or psychiatric admissions (SP19). |
|  |  | Information sharing | Barriers in information sharing within the organizations | CLBC relies on other systems to create access pathways to ours, but those systems don't know sometimes that we're relying on them (DM04). |
|  | Current solutions | Organizational solutions | Actions taken by the organizations to reduce the challenges faced by families | It’s our effort to try and increase literacy on these services within our organization (SP02). |
|  | Services | Services offered | Services available for the youth and families | CLBC engages with providers, individuals, and families, and incorporates emerging service needs into strategic planning (DM01). |
|  |  | Service connections | Positive outcomes for families from having connections within services | An employment pilot helps high school students with disabilities enter the job market, reducing reliance on day services and fostering community connections (SP04). |
|  |  | Service awareness | Awareness about the services among families | Ongoing education is needed since, as a smaller population, we’re not always a primary consideration for other systems (DM04). |
| Exosystem: Structural Barriers to Equitable Service Access | Available resources | Funding | Funding eligibility and availability | CLBC has a finite budget [..]. So we try to manage within the parameters and then prioritize individuals who have the greatest needs (DM02). |
|  |  | Geographical location | Support personal for the families in remote locations | Not just rural that you can drive to, but remote where it's fly in, fly out for part of the year and there may or may not be any services in the community at all (DM04). |
|  | Challenges in transition | System related | Challenges that families face in transition at system level | In rural and remote communities, limited infrastructure creates disparities in eligibility and access to services (DM10). |
|  | Policy | Eligibility criteria | Eligibility to transition for adult services in policy level | You (either) meet the threshold requirements and have a developmental disability, autism or FASD you are then eligible to receive services from CLBC (DM08). |
|  |  | Decision making | How the decisions about eligibility of the families are made | Individuals with developmental disabilities may be ineligible if they don’t meet the cognitive impairment threshold, which often frustrates families (DM04). |
|  |  | Triage | How does an organization assess who needs urgent services | We try to manage within the parameters and then prioritize individuals who have the greatest needs. But given the limited resources, it may mean that someone else may have to wait a bit longer (DM02). |
| Macrosystem: Societal and Cultural Influences | Cultural aspects | Immigration status | How immigration status affects the transition process | Among some immigrant families, cultural beliefs about intellectual disability can create shame and hinder support (SP04). |
|  |  | Trust | Trust related barriers about the system | Some families face cultural barriers, viewing diagnosis and systemic involvement as unnecessary or unwanted (DM04). |
|  |  | Perspectives of youth and family | Perspectives that families have about disability | It depends on your cultural background and maybe those perspectives on disability and sometimes that might prevent you from accessing (SP08). |
|  | Privilege | Discrimination | Barriers related to privilege when accessing services | It's those families around the individuals who have the capacity to do that, and quite honestly, who have probably a little bit of privilege. Whether it's financial, racial, intellectual, whatever it is, there's some level of discrimination that enables that to not happen (DM2). |
|  |  | Socio-economic status | Facilitators related to socioeconomic status when accessing services | People with intellectual disabilities who are born into affluent privileged families have by far a better situation, receive better services, have better housing (SP04). |
| Chronosystem: Influence of Time and Policy Shifts | Changing needs | Needs of youth and families | What are the service needs that are changing for the families? | Kids that are transitioning into adulthood, we're seeing changing needs as we become more aware of the strengths and contributions that neurodiverse people can make to society (SP08). |
|  |  | Needs of inclusivity | What are the service needs that are changing in society? | I think a much greater need and right to just be part of the community. So to get away from the more segregated models of support to much more inclusive models. So I think that's one need that needs to be addressed by services changing (SP04). |
|  |  | Needs through systems | What are the service needs that are changing in organizational level? | There are new opportunities, new challenges, new service needs (DM2). |
| Policy implications | Person-centered transition planning |  | Individualized approaches that ensure families have a dedicated transition plan tailored to their needs | Case management would be a helpful thing [..] you need that one person to help hold the story as they go from child and youth to adults (SP02). |
|  | Stronger collaboration between systems |  | The need for improved coordination and communication among organizations | It is about how, when and who we engage with [and] pursue it more actively than historically (DM04). |
|  | Gaps in regional coordination |  | How services are organized and connected across regions | CLBC has missed that middle gap[..] we need to be in the local networks, but also in the regional network (DM04). |
|  | Outreach and culturally responsive supports |  | Efforts to raise awareness of available services and provide supports that are accessible | It’s important that we are making sure organizations are aware of our services (DM01). |
|  | Resourcing |  | Expanding system capacity | It would be great if we could increase the ability to support adults (SP08). |
|  | Emerging needs |  | Evolving barriers such as co-occurring conditions | It is important to address significant developmental changes, you're transitioning away from an environment that perhaps you've become used to, such as substance use (DM10). |
