# Supplementary material for: Service access for youth with neurodevelopmental disabilities transitioning to adulthood: service providers’ and decision-makers’ perspectives on barriers, facilitators and policy recommendations
Source: Front Public Health. 2025 Nov 6;13:1612509. doi: 10.3389/fpubh.2025.1612509 (PMC12631288; doi:10.3389/fpubh.2025.1612509)
Supplement: SUPPLEMENTARY DATA SHEET 1 — Interview guide. [file Data_Sheet_1.docx]

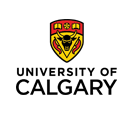


**Interview Guide**

**Background**

Researchers from the University of Calgary, including the School of Public Policy, Faculty of Social Work and Cumming School of Medicine, have partnered with youth and family advisors and government ministries in Alberta and British Columbia to design a project named ACCESS *(Assessing the Continuum of Care and Eligibility for Services and Supports for Children with Neurodevelopmental Disabilities and their Families).* The project aims to identify pathways and disparities in accessing existing health, education and social services for Youth with Neurodevelopmental Disabilities (NDD) and their families. We further seek to understand how different pathways and disparities may influence outcomes for youth across childhood, adolescents and early adulthood. The findings of this study will inform policy and program changes by working collaboratively with decision-makers to enhance services for youth to improve outcomes.

**About the Interview Guide**

The interview will ask questions about your role, inclusion and exclusion criteria for the service users (families/ children/youth) and barriers and facilitators to accessing services.

**Interview Discussion Points**

**1. Background and Role/Warm-up**

*Question:* Can you please tell me about your role(s) with the Uniti organization?

Probe 1: For how long have you been working with Uniti organization?

Question: What kind of service does your organization offer to young adults with neurodevelopmental disabilities (NDD) and their families?

Question: Can you please tell us with how many young adults with neurodevelopmental disabilities (NDD) and families have you worked with in the last year?

**2. Service Awareness**

*Question:* How do young adults and their families hear about the services offered by your organization under CLBC?

*Potential probes:* How are young adults and their families referred to your program? What are the typical access points to your service?

**3. Eligibility Criteria**

Question: Are you aware of what has made it harder for young adults with NDD and their families to be eligible for CLBC services?

Question: Are you aware of what has made it easier for youth with NDD and their families to be eligible for your services?

Question: Have the eligibility criteria (with respect to CLBC services) changed over time for young adults with NDD and their families? In your opinion, what has influenced changes in eligibility criteria?

Questions: Whom do you talk to if you feel a young adult and family need services under CLBC despite not meeting eligibility criteria?

**4. Access to Services**

*Question:* What types of barriers might young adults and families face in accessing services under CLBC?

Question: Are there some types of young adults and families that face more barriers in accessing CLBC services than others? If yes, what types of families are more/less vulnerable?

Probe – Are there any barriers that could be based on gender?

Probe – Are there any barriers that could be based on socio-economic or demographic status?

Probe: What could be done differently to help address the barriers to young adults and families accessing CLBC services?

Question: Are there any facilitators that help young adults and their families to access services under CLBC?

For transitioning (adolescence/adult youth) cohort: Are there any factors which could contribute to young adults and families losing services during the transfer of care/transition period?

Probe: What could be done differently to address these factors?

Question 5: How do you think services provided by CLBC influence quality of life in the young adults and the families that your organization serves?

**5. Changing Needs**

1. Question: Are there any changing needs of young adults and families over time, during transition to adolescence or adulthood. Changing needs, for example, could be: functional changes, developmental changes.
2. Question: Are there any program/service guidelines to address changing needs of youth and families over time?

**6. Waitlist**

Question: How long do young adults and families wait for an intake in CLBC services?

Question: How long do young adults and families wait to receive services?

Probe: Are you aware if any steps are taken by CLBC to reduce the wait times,

Question: What could be done to reduce the wait time? What could be done to help families while on the waitlist?

**7. Family Function**

Question: How do you assess if young adults and families need urgent services?

Question: How do you triage supports for young adults and families?

Potential probes: Is there any criteria? Is it first come first serve or needs based?

Question: Is there any training for you and your colleagues about working with under-represented populations?
